# Assessing Sleep and Mental Health Disorders in COPD Patients During Severe Exacerbations

**DOI:** 10.2174/0118743064410899251013073201

**Published:** 2026-03-25

**Authors:** Tanisha Dighe, Shyam Subramanian, Sneha Reddy, Munish Sharma, Salim Surani

**Affiliations:** 1 Department of Pulmonary Critical Care, Sutter Gould Medical Foundation, California, USA; 2 Department of Pulmonary, Critical Care and Sleep Medicine, Sutter Health, California, USA; 3 Department of Biology, The University of Texas, Austin, USA; 4 Department of Pulmonary and Critical Care, Baylor Scott & White Medical Center - Temple, USA; 5 Department of Medicine, Texas A&M University, College Station, USA

**Keywords:** Sleep, Insomnia, Depression, COPD, Mental health, Anxiety

## Abstract

**Introduction:**

Acute exacerbations of Chronic Obstructive Pulmonary Disease (COPD) frequently result in hospitalization of the patients. The sleep health of patients admitted to the hospital with COPD exacerbations may be overlooked. The objective of this study was to assess and define the prevalence of sleep and mental health complaints among patients admitted with acute exacerbations of COPD.

**Methods:**

In this prospective study, patients admitted with an episode of COPD exacerbation at a local community hospital were given a list of questionnaires pertaining to sleep and mental health. These questionnaires included the Beck Depression Inventory (BDI-II), the Functional Outcomes of Sleep Questionnaire (FOSQ), the Epworth Sleepiness Scale (ESS), and the Pittsburgh Sleep Quality Index (PSQI). Questionnaires were administered in a stable, steady state on discharge.

**Results:**

53 patients filled out the questionnaires. 50.9% of patients reported poor sleep quality with scores indicative of chronic insomnia, and 41.2% of patients reported excessive daytime sleepiness on the PSQI. 64% of patients indicated abnormal total scores (<18) on the FOSQ, with 84.3% of patients reporting severe impairment in social outcomes. Clinical depression BDI scores >9) was seen in 73% of patients.

**Conclusion:**

Our results indicate a significant prevalence of sleep and mental health comorbidities in patients hospitalized for acute COPD exacerbations and highlight the need for screening tools and clinical interventions to reduce the burden of these comorbidities.

## 1 INTRODUCTION

Chronic Obstructive Pulmonary Disease (COPD) is one of the main health problems worldwide. COPD is estimated to affect approximately 200 million people globally and is estimated to contribute to nearly 10% of the global disease burden [1, 2]. COPD is primarily characterized by chronic, irreversible airflow limitation. There is a high prevalence of systemic comorbidities, including sleep and mood disorders such as depression and anxiety, amongst others [3, 4]. These comorbidities can exacerbate the clinical course of COPD and lead to an overall reduction in Quality of Life (QOL). These comorbidities also impair physical functioning and increase healthcare utilization [5, 6]. The pathophysio-logical mechanisms linking COPD with sleep and mood disturbances are multifactorial. These include chronic hypoxemia, hypercapnia, sleep-related hypoventilation, effects of nicotine withdrawal, and adverse effects of medications like bronchodilators and corticosteroids (Table **1**) [6, 7].

Acute exacerbation of COPD is primarily characterized by worsening of the respiratory symptoms leading to the need for medical intervention. It is estimated that annually, around 50% of patients with COPD suffer from exacerbation episodes and remain a leading cause of morbidity, hospitalization, and mortality [8, 9]. COPD exacerbations cause a decline in lung function and cause significant psychological and physical burdens to patients. It has been found that poor sleep quality is associated with a higher risk of further COPD exacerbations, increased dyspnea, and decreased exercise tolerance [10]. The impact of sleep and mood disorders in a stable COPD is well known, but their effects during acute exacerbation of COPD (AECOPD) are not well studied.

Our study aims to address this critical gap in knowledge by quantifying the prevalence of sleep disturbances and mood disorders in patients hospitalized for AECOPD. Using validated tools, we assess sleep quality, daytime sleepiness, and depression to characterize the burden of these comorbidities. Understanding these trends may help develop better strategies to manage COPD by addressing both respiratory symptoms and mental health concerns.

## 2 MATERIALS AND METHODS

This prospective, single-center study was conducted at a community hospital and included adult patients admitted for moderate acute exacerbations of chronic obstructive pulmonary disease (COPD). The patient admitted had the prior diagnosis of COPD as an outpatient based on spirometry and clinical vignette. Participants were identified based on clinical presentation and a confirmed diagnosis of COPD exacerbation, defined as a sudden worsening of respiratory symptoms requiring medical intervention. AECOPD was diagnosed by the treating physician based on a combination of clinical history (increased dyspnea, sputum volume, or purulence), physical examination findings, and radiologic and laboratory evaluations. Patients with primary diagnoses of pneumonia (defined by radiographic consolidation), congestive heart failure (based on echocardiographic findings or elevated BNP with compatible clinical presentation), or asthma (based on prior diagnosis and bronchodilator reversibility) were excluded to minimize diagnostic confounding. There were patients with overlap between COPD and Asthma in the study. Inclusion criteria comprised adult patients aged 18 years or older hospitalized for an acute exacerbation. Exclusion criteria included evidence of pulmonary conditions other than COPD (*e.g.*, interstitial lung disease, pulmonary embolism, or lung cancer), a history of radiographically suspicious pulmonary nodules, or a Body Mass Index (BMI) greater than 34, as obesity can independently influence sleep and mood parameters, history of insomnia, obstructive sleep apnea, depression, psychosis and post-traumatic stress disorder. Following eligibility screening and application of exclusion criteria, 53 patients were included in the study. 53 patients were included in the study from July 1^st^ 2018 to December 31^st^ 2018.

Approval for this study was obtained from the Institutional Review Board (IRB) of Corpus Christi Medical Center, Texas, USA, and written informed consent was obtained from all participants. Data collection occurred on the day of discharge, as patients were considered to be in a clinically stable state, thereby minimizing the acute impact of exacerbations on subjective assessments. Participants were administered a series of validated self-reported questionnaires to evaluate sleep quality, daytime sleepiness, functional impairments, and depressive symptoms.

The Beck Depression Inventory-II (BDI-II) was utilized to assess depressive symptoms over the preceding two weeks. This 21-item instrument employs a 4-point Likert scale (0–3) and provides total scores categorized as minimal/no depression (0–9), mild to moderate (10–18), moderate to severe (19–29), and severe (30–63). Items specific to sleep disturbances, such as difficulty initiating or maintaining sleep, are particularly relevant in COPD (11). To evaluate the impact of sleep disturbances on daily functioning, the Functional Outcomes of Sleep Questionnaire (FOSQ) was administered. This 30-item instrument examines five domains: activity level, vigilance, intimacy and sexual relationships, general productivity, and social outcomes. Responses are rated from “extreme difficulty” to “no difficulty,” with lower scores indicating greater functional impairment caused by excessive sleepiness (12). The Pittsburgh Sleep Quality Index (PSQI) was used to assess subjective sleep quality across seven domains: subjective sleep quality, sleep latency, sleep duration, habitual sleep efficiency, sleep disturbances, use of sleep medications, and daytime dysfunction. Each domain is scored on a scale of 0–3, with the global PSQI score (range: 0–21) reflecting overall sleep quality; higher scores indicate poorer sleep quality (13). Excessive Daytime Sleepiness (EDS) was assessed using the Epworth Sleepiness Scale (ESS). This 8-item questionnaire evaluates the likelihood of dozing off during various sedentary activities, such as watching television or sitting quietly. Responses range from 0 (“would never doze”) to 3 (“high chance of dozing”), with total scores ranging from 0 to 24. A score of 11 or higher was considered indicative of significant EDS (14).

Descriptive statistics were calculated to summarize demographic and clinical data. Continuous variables, such as age, BMI, and questionnaire scores, were expressed as means with standard deviations, while categorical variables were presented as frequencies and percentages. Between-group comparisons were performed using independent t-tests for continuous variables and chi-square tests for categorical variables. A *p*-value of less than 0.05 was considered statistically significant. All statistical analyses were conducted using Microsoft Excel and SPSS version 25 for Windows (IBM Corp., Armonk, NY).

## 3 RESULTS

This study included 53 patients admitted for acute exacerbations of chronic obstructive pulmonary disease (COPD). The majority of the study population (92.5%) had a diagnosis of COPD alone, while a small proportion presented with features of COPD and asthma overlap syndrome. The mean age of participants was 65 ± 14 years, and the cohort consisted of 52.8% females (n=28) and 47.2% males (n=25), reflecting a near-equal gender distribution.

### 3.1 Beck Depression Inventory-II (BDI-II)

The BDI-II was utilized to assess the severity of depressive symptoms within the study population. Clinical depression, defined as a BDI-II score >9, was observed in 73% of the participants. Notably, 39.6% (n=21) exhibited moderate to severe depression (BDI-II score 19–29), and 13.2% (n=7) reported scores indicative of severe depression (BDI-II score ≥30). Depression prevalence did not differ significantly between genders, with 43.4% of males and 39.6% of females reporting clinically significant depressive symptoms. The analysis of specific BDI-II items revealed notable trends. Anhedonia, reported by 70.5% (n=39) of participants during exacerbation, underscores the emotional burden in this acute phase. While causality cannot be established and mood symptoms may be transiently influenced by steroid use, these findings highlight a need for psychological support during exacerbations. Additionally, 29.4% of patients indicated waking up 1–2 hours earlier than usual, with difficulty returning to sleep, which reflects underlying sleep disruption associated with depressive symptoms. Functional impairments (question 15), particularly difficulty in maintaining daily routines, were reported in 29.4% of participants, underscoring the overlap between physical debilitation and depressive symptomatology in this population.

### 3.2 Functional Outcomes of Sleep Questionnaire (FOSQ)

The Functional Outcomes of Sleep Questionnaire (FOSQ) revealed significant sleep-related functional impairments in the cohort. The mean FOSQ score for the group was 15.4 ± 6.12, indicating substantial difficulty across various functional domains. A total of 64.2% (n=34) of participants had abnormal FOSQ scores (<18), reflecting clinically relevant impairments in daytime functioning. Among those with abnormal scores, 59.4% (n=19) were males and 40.6% (n=13) were females. Domain-specific analysis of FOSQ revealed that social outcomes were the most severely affected, with 84.3% of patients reporting significant impairment (scores ≤1). This suggests a profound disruption in social engagement, possibly exacerbated by sleep disturbances, daytime sleepiness, and mood disorders. Additionally, general productivity was severely reduced in 27.4% of participants, while 41.1% reported an absence of, or a significantly impaired, intimate relationship, highlighting the far-reaching impact of poor sleep quality on emotional and interpersonal functioning. Vigilance, an essential component of cognitive functioning, was another domain significantly affected, with 33.3% of patients reporting extreme difficulty (scores <1) in maintaining alertness during daily activities.

**Fig. (1):**
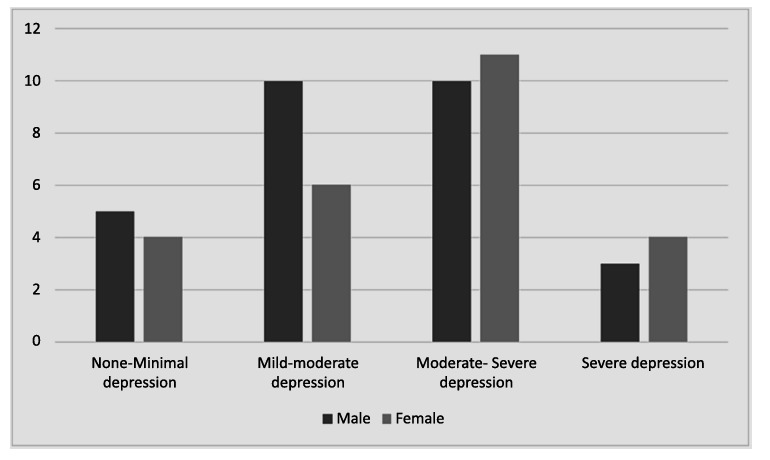
Overview of FOSQ and PSQI in COPD patients with acute exacerbation ((n) indicates prevalence of abnormal results).

Notably, there was a significant overlap between depression and poor functional outcomes: 78.1% of patients with abnormal FOSQ scores also exhibited at least mild-to-moderate depression, reflecting the interconnected nature of sleep disturbance, functional impairment, and mood disorders (Fig. **1**).

### 3.3 Pittsburgh Sleep Quality Index (PSQI)

The Pittsburgh Sleep Quality Index (PSQI) was administered to evaluate subjective sleep quality. Poor sleep quality, defined as a PSQI global score ≥5, was reported by 50.9% of participants, indicating a high prevalence of sleep disruption. Sleep latency, the time taken to initiate sleep, was markedly prolonged, with an average latency of 65 minutes. Specifically, 74.5% of participants reported taking 30 minutes or longer to fall asleep, with 25.4% experiencing severe delays (>60 minutes). Despite the prolonged sleep latency, the majority of patients (60.8%) reported good sleep efficiency, defined as >85%. Sleep duration was notably variable: 23.5% reported ≤6 hours of total sleep time, while only 39.2% reported>7 hours. Importantly, 68.6% of patients indicated that they had not used any sedatives or hypnotics, reflecting either patient reluctance to use pharmacologic interventions or clinician hesitancy to prescribe them due to concerns regarding respiratory depression. Mild to moderate daytime disturbances related to sleep, such as waking up early, temperature-related discomfort, dreams, and breathing difficulties, were reported by 96.1% of participants. Daytime dysfunction, including trouble staying awake during activities such as driving, eating meals, or social interactions, was noted in all patients, although the majority (96.1%) rated these disturbances as mild to moderate on the scale (Fig. **1**).

### 3.4 Epworth Sleepiness Scale (ESS)

Excessive daytime sleepiness (EDS), as assessed using the Epworth Sleepiness Scale (ESS), was prevalent in this cohort. A total of 41.2% of participants had ESS scores >10, consistent with clinically significant EDS. Severe daytime sleepiness, defined as an ESS score >16, was observed in only 5.7% (n=3) of patients. While severe EDS was relatively uncommon, the high prevalence of moderate EDS underscores its impact on daily functioning and highlights the need for comprehensive sleep assessments in patients with acute COPD exacerbations.

## 4 DISCUSSION

### 4.1 Sleep and COPD - Sleep Quality

There are multiple factors that affect the relationship between COPD and sleep disorders. Disease physiology, comorbidities, and environmental factors all seem to play a role. Quality of sleep is impacted by chronic hypoxemia, systemic inflammation, hypercapnic state, and medications used to treat COPD, such as steroids and bronchodilators. Habitual nicotine use can also interfere with normal sleep architecture [11-18].

Significance of Results: It was evident in our study that around 50.9% of patients reported poor quality of sleep with Pittsburgh Sleep Quality Index (PSQI) scores indicating moderate to severe chronic insomnia. Although 84.4% of patients reported preserved sleep duration of >5 hours, sleep latency was abnormal in 74.5%, with 25.4% experiencing severe delays. Similar findings were reported by Budhiraja *et al*., who found that reduced sleep efficiency (<85%) affected 44% of COPD patients [15]. In contrast, our study showed slightly lower sleep efficiency, with 39.2% reporting reduced quality. This discrepancy may be due to differences in the settings of the study or population characteristics.

Nocturnal symptoms such as cough and dyspnea, along with hypoxia dusrupt the ability to initiate and maintain sleep in patients with COPD. These mechanisms also lead to fragmented sleep, reduced slow-wave sleep, and an increased time spent in lighter, non-REM stages [7]. These abnormalities can have further downstream effects on daytime function, emotional well-being, and quality of life of patients.

### 4.2 Daytime Sleepiness and COPD

Excessive Daytime Sleepiness (EDS) is a commonly reported symptom in patients with COPD. It can impair daily functioning, productivity, and overall quality of life [7]. Our study found that 41.2% of patients reported abnormal daytime sleepiness on the Epworth Sleepiness Scale (ESS). This result is similar to previous literature and highlights that EDS is a common but often underrecognized symptom in COPD. This could be more relevant during acute exacerbations when nocturnal symptoms may worsen [19]. A significant confounding factor here may be the presence of co-morbid sleep apnea in patients with COPD. We did not perform screening for OSA, but previous studies have reported that OSA prevalence ranges from 16% to 66% in COPD patients, and this could have been a contributing factor in our study as well [19]. Coexistence of COPD and OSA (often termed as an overlap syndrome) exacerbates both daytime and nocturnal symptoms, but this was beyond the scope of our study.

There are multiple factors causing EDS in COPD patients: nocturnal hypoxemia, hypercapnia, dyspnea, and frequent awakenings due to cough or discomfort disrupt sleep continuity, and reductions in overall sleep quality can all lead to EDS in COPD [7]. Additionally, systemic inflammation during acute exacerbations and altered respiratory mechanics likely exacerbate sleep fragmentation, further contributing to daytime sleepiness [15].

### 4.3 Mood Disorders, Functional Outcomes, and COPD

Mental health comorbidities are among the most pervasive yet underrecognized issues in COPD patients. In our study, clinical depression (Beck Depression Inventory-II score >9) was reported in 73% of participants, indicating a significant psychological burden. This prevalence aligns with findings from other studies, where rates of depression in COPD patients have ranged from 40% to 75%, depending on disease severity and study setting [20, 21]. However, most of those studies are conducted in outpatient settings. In a prior study in an inpatient setting using the Hospital Anxiety and Depression Scale (HADS) in Spain, the prevalence of probable depression was estimated to be 67.7%, and this appears consistent with the data provided in the study [22].

The study also demonstrated a significant impact of poor sleep and depression on functional outcomes, as measured by the Functional Outcomes of Sleep Questionnaire (FOSQ). A total of 64% of patients reported abnormal FOSQ scores, suggesting severe impairment in key functional domains. Notably, 84.3% reported deficits in social functioning, likely reflecting the combined effects of sleep disruption, daytime fatigue, and mood disorders. These deficits exacerbate social isolation and physical inactivity, which are common in COPD patients and further reduce overall quality of life.

The interplay between COPD, sleep, depression, and poor functional outcomes is well-documented. Depression is associated with higher healthcare utilization, increased readmission rates, and worse adherence to treatment regimens [21]. Furthermore, sleep disturbances and depression are bidirectionally related, each exacerbating the other [23]. Addressing these issues in parallel through targeted interventions may help mitigate their combined impact on patient outcomes.

### 4.4 Study Limitations

Our study results provide valuable insights. However, we must acknowledge the limitations of the study as well. First, this study relied on self-reported questionnaires, which, while validated, remain subjective. Objective assessments such as polysomnography or actigraphy could provide a more robust measure of sleep quality. Second, the study's sample size was smaller (n=53). The sample size was obtained from a single community hospital. Hence, the findings of this study can not be generalized. We had a higher proportion of female patients (52.8%), which is atypical for a COPD patient cohort, which generally consists of a higher male patient population. This could be a reflection of selection bias or may represent a regional smoking pattern. The sample size precluded subgroup analyses by sex, and as a result, sex-adjusted analyses could not be performed. These factors should be considered when interpreting the results. Larger multicenter studies are needed to generalize the findings of the study. Assessments were conducted at discharge to ensure clinical stability. However, it is acknowledged that this timing may reflect nonspecific effects of hospitalization (*e.g.*, sleep disruption and stress) rather than changes related solely to exacerbation. Additionally, the absence of a control group of hospitalized patients without COPD limits the ability to attribute these findings exclusively to COPD rather than to the general effects of hospitalization. Inclusion of clinical variables such as hypoxia, ICU admission, or the use of non-invasive ventilation or intubation could have helped avoid confounding of the association between disease severity and sleep or mental health-related outcomes. Additionally, while the PSQI assesses sleep quality over the past month, it may not accurately capture chronic insomnia, which requires symptoms for at least three months. Future studies should incorporate these clinical variables and validated tools for chronic insomnia. Lastly, although the focus was on sleep quality and mental health during hospitalization, assessing the longitudinal impact of these comorbidities following discharge would be valuable.

Identifying whether interventions targeting sleep or mental health improve long-term COPD outcomes could help refine management strategies.

### 4.5 Clinical Implications and Future Directions

This study identified a significant gap in the routine assessment of sleep quality and mental health among patients hospitalized with acute exacerbations of COPD (AECOPD). Simple, validated screening tools such as the Pittsburgh Sleep Quality Index (PSQI), Epworth Sleepiness Scale (ESS), and Beck Depression Inventory-II (BDI-II) can be readily incorporated into clinical practice to identify patients at risk. Early identification of mood disorders and sleep disturbances may facilitate timely intervention. Non-pharmacological approaches, including cognitive behavioral therapy for insomnia (CBT-I) and targeted treatments for depression and anxiety, may serve as appropriate interventional strategies.

The high prevalence of daytime sleepiness observed in this study highlights the need to screen for comorbid obstructive sleep apnea (OSA). Formal sleep studies targeted toward high-risk patients, along with timely initiation of continuous positive airway pressure (CPAP) therapy, may contribute to improvements in respiratory symptoms and overall sleep quality.

Future studies should aim to integrate interventions addressing sleep quality, mental health, and functional impairments to better evaluate their impact on COPD-related morbidity and mortality. A multidisciplinary care model involving pulmonologists, sleep specialists, and mental health professionals may further enhance patient outcomes by comprehensively addressing these interconnected factors.

## CONCLUSION

Acute exacerbation of COPD is one of the main causes of hospital admissions. Patients with COPD are at higher risk of sleep disturbances and mental health issues. During the hospital stay of patients with AECOPD, it is important not only to treat respiratory symptoms but also to identify and manage comorbidities that may exacerbate the course of COPD. Increased awareness and access to screening tools can help healthcare providers recognize subtle or masked symptoms of sleep and mental health disturbances in this patient population. By implementing effective interventions, healthcare providers can improve the quality of life for COPD patients and potentially reduce the burden of COPD exacerbations. Healthcare providers can take significant steps toward the overall well-being of COPD patients by addressing their sleep and mental health care.

## DECLARATION

An abstract of this study was presented at the American College of Chest Physicians (CHEST) Annual Meeting and published as a supplement in the *CHEST* journal [24].

## Figures and Tables

**Table 1:** Pathogenesis of sleep disorders in COPD.

**Sleep Disorder**	**Etiologic Factors**	**Increased Risk of**
Insomnia	-Respiratory symptoms -Nicotine use/withdrawal -Increased WOB, -hypoxia -Increased sympathetic activity -Comorbid anxiety and depression -Primary sleep disorders -Medications: theophylline, beta agonists, steroids	-Hypertension -Diabetes -Atherosclerosis -Myocardial infarction -Mortality
Sleep-related hypoxemia	-Increased airway resistance -reduced TV, FRC, minute ventilation -hyperinflation and flattening diaphragm -Diminished tone of accessory respiratory muscles	Hypertension Pulmonary hypertension Right ventricular dysfunction Cardiac arrhythmias Nocturnal death
Sleep hypoventilation		
OSA Central sleep apnea	-Chronic Steroid use -Increased airway edema from cor pulmonale -Decreased exercise capacity > obesity -Muscle weakness > increased airway collapsibility -presence of GERD, allergies, obesity, smoking	-cardiac dysrhythmias -severe pulmonary hypertension -right heart failure.
Restless leg syndrome	hypoxemia- hypercapnia induced dopamine imbalance Co-existing iron deficiency, renal failure	depression, anxiety, and panic disorder

## Data Availability

The data supporting the findings of the article will be available from the corresponding author [S.S] upon reasonable request.

## LIST OF ABBREVIATIONS

OSA: Obstructive Sleep Apnea
PSQI: Pittsburgh Sleep Quality Index
FOSQ: Functional Outcomes of Sleep Questionnaire
CPAP: Continuous Positive Airway Pressure
IRB: Institutional Review Board

## STANDARDS OF REPORTING

STROBE guidelines were followed.
